# Dynamics of Circulating Brain-Derived Neurotrophic Factor and Selenoprotein P in Subacute Stroke Patients Undergoing High-Intensity Interval Training-Based Neurorehabilitation: A Pilot Observational Study

**DOI:** 10.3390/neurolint18080154

**Published:** 2026-08-20

**Authors:** Hunor-Pál Fodor, Beáta Albert, Pál Salamon

**Affiliations:** 1 Faculty of Natural Sciences, University of Pécs, Ifjúság útja 6., 7624 Pécs, Hungary; 2 Faculty of Economics, Socio-Human Sciences and Engineering, Sapientia Hungarian University of Transylvania, 530104 Miercurea Ciuc, Romania

**Keywords:** brain-derived neurotrophic factor, Selenoprotein P, subacute stroke, high-intensity interval training, neuroplasticity, peripheral biomarkers, central sink hypothesis, pilot clinical trial

## Abstract

**Background/Objectives:** Brain-derived neurotrophic factor (BDNF) and antioxidant networks mediated by Selenoprotein P (SEPP1) are core drivers of structural neuroplasticity and blood–brain barrier integrity, yet their co-regulatory behavior during subacute stroke neurorehabilitation remains poorly understood. This pilot study quantified concurrent changes in serum BDNF and SEPP1 during rehabilitation. **Methods:** Sixteen subacute post-stroke patients with mild stroke severity were assigned to an intensive multi-modal neurorehabilitation protocol incorporating high-intensity interval training (Treated, *n* = 7) or standard care (Control, *n* = 9); the biomarker sampling window averaged 73.4 days. Fasting venous blood was collected at baseline and post-intervention and analyzed by ELISA. **Results:** BDNF changes (ΔBDNF) differed significantly between arms (U = 57.0, *p* = 0.0081): the Treated cohort showed a uniform decrease (mean Δ: −0.240 ± 0.103 ng/mL), while Controls showed stabilization or a slight increase (mean Δ: +0.118 ± 0.339 ng/mL). ΔBDNF and ΔSEPP1 were significantly, positively correlated across the cohort (ρ = 0.596, *p* = 0.015). **Conclusions:** The BDNF decline in the Treated group is consistent with the “central sink” hypothesis, but may more plausibly reflect stress/cortisol-mediated suppression induced by the high training intensity, contrasting with increases typically reported after moderate-intensity subacute-phase exercise. The collinear coupling with SEPP1 suggests a link between neurotrophic synthesis and antioxidant buffering during post-stroke tissue remodeling, though this correlational finding does not by itself establish a single, coordinated mechanism.

## 1. Introduction

Ischemic stroke and severe transient ischemic attacks (TIAs) represent leading causes of persistent adult disability worldwide, triggering specialized neuropathological cascades that result in long-term motor, sensory, and cognitive deficits. The subacute phase following stroke represents a critical window of heightened endogenous neuroplasticity, during which structured, activity-dependent rehabilitation can substantially influence the trajectory of cortical reorganization and white-matter remodeling [1]. At the center of these cellular adaptations is brain-derived neurotrophic factor (BDNF), a premier neurotrophin that regulates synaptic long-term potentiation (LTP), dendritic branching, and neuronal survival within the peri-infarct zone [1,2].

Epidemiological and clinical observations demonstrate that stroke patients frequently exhibit altered circulating BDNF concentrations relative to age-matched healthy controls, both in the acute phase and, more specifically, among those surviving into the chronic post-stroke phase [3,4,5]. However, considerably less is known about BDNF dynamics during the earlier subacute phase, when endogenous neuroplastic and rehabilitative potential is greatest. This systemic deficit represents a critical biochemical bottleneck that can impede effective neurorehabilitation. Physical exercise and targeted sensorimotor training have been shown to overcome this limitation by stimulating central BDNF expression. This process occurs via calcium-dependent intracellular signaling cascades, notably involving calmodulin-dependent protein kinase II (CaMKII) activation and the subsequent up-regulation and phosphorylation of the transcription factor cAMP response element-binding protein (CREB) [6,7]. Advanced clinical models, including high-intensity interval training (HIT) and structured aerobic conditioning protocols, have highlighted the therapeutic value of physical loading in augmenting peripheral biomarker availability and driving downstream structural neurogenesis [7,8,9].

Nevertheless, tracking these neuroplastic transformations through peripheral venous sampling presents an array of complex, non-linear findings. While acute physical exertion is known to induce transient spikes in circulating neurotrophin levels due to platelet degranulation [6,7], the systemic long-term adaptations of baseline serum BDNF across a multi-week rehabilitative window are often variable. Furthermore, neuroplasticity does not occur in a metabolic vacuum; it requires a highly responsive antioxidant defense infrastructure to buffer the reactive oxygen species (ROS) generated during intensive neurorehabilitation. Selenoprotein P (SEPP1), the primary extracellular glycoprotein responsible for systemic selenium transport to the central nervous system, plays a crucial role in maintaining redox homeostasis, safeguarding blood–brain barrier structural integrity, and protecting newly forming synaptic networks from oxidative damage. Despite its clear physiological relevance, the co-regulatory behavior and bidirectional cross-talk linking circulating neurotrophins to selenium-dependent antioxidant networks have not yet been evaluated in a subacute stroke population.

To address this knowledge gap, this pilot study was designed as a targeted clinical Communication to investigate the concurrent alterations of serum BDNF and SEPP1 levels in subacute stroke patients undergoing a structured, multi-modal neurorehabilitation program compared to conventional-care controls. We hypothesized that active physical and functional training would induce distinct biomarker shifts reflecting central tissue extraction and that these changes would be tightly linked to systemic antioxidant responses.

## 2. Materials and Methods

Participant Enrollment and Clinical Allocation: A total of 16 subacute post-stroke patients with mild stroke severity, presenting with persistent upper-extremity motor hemiparesis, were recruited between March and July 2024 for this pilot clinical evaluation. The study population was allocated into two parallel observational groups: the Neurorehabilitation Group (Treated cohort, *n* = 7) and the Conventional Management Group (Control cohort, *n* = 9). Group allocation was not randomized; a cardiologist evaluated each patient’s cardiovascular suitability for the structured high-intensity aerobic protocol, and only patients judged suitable were assigned to the Treated cohort, while the remaining patients received conventional care as the Control cohort. The baseline clinical characteristics (including biological sex, chronological age, primary cerebrovascular diagnosis, and active comorbidities) were thoroughly documented at intake. The study was conducted in strict accordance with the ethical guidelines of the Declaration of Helsinki and received formal validation and approval from the Institutional Review Board of Sapientia Hungarian University of Transylvania (Protocol Code: 1/2023; Date of Approval: 3 January 2023). Prior to baseline profiling, all participants or their legally authorized representatives provided written informed consent.

Neurorehabilitation Protocol: Patients assigned to the Treated cohort underwent a structured 8-week program (24 sessions total; 3 sessions per week) combining long-interval high-intensity interval training (HIT) with individualized physiotherapy. Each session began with 6–8 min of joint mobilization exercises for the major joints, followed by 30 min of treadmill-based long-interval HIT and 30–35 min of physiotherapy. The HIT protocol consisted of a 5-min warm-up walk at 50–60% of peak-oxygen-uptake-associated heart rate (VO_2_peak-HR), three 4-min intervals of intense walking at 85–95% VO_2_peak-HR, interspersed with two 4-min active-recovery walking intervals at 50–60% VO_2_peak-HR, and a 5-min cool-down walk at 50–60% VO_2_peak-HR, following the long-interval HIT framework described for post-stroke rehabilitation [10]. This approach was selected because HIT provides more potent, intermittent hemodynamic stimuli than continuous moderate-intensity training, which may enhance shear stress, endothelial adaptation, and cardiovascular stimulation, potentially yielding greater improvements in peak oxygen uptake and vascular function in stroke populations [10]. The physiotherapy component comprised joint mobilization exercises to restore range of motion (8 min), adapted ballistic strength training targeting gait speed and stride length (12–15 min), and balance/postural control exercises, including static and dynamic balance, reactions to external perturbations, center-of-mass control, turning, and functional standing tasks such as stair negotiation (10–12 min). The cumulative treatment window varied from 74 to 102 days (mean duration: 82.29 ± 10.87 days) based on individualized clinical progression tracks. Conversely, patients in the Control cohort did not participate in the structured physical rehabilitation program, maintaining their standard daily routines and conventional medical regimens over an observation window ranging from 49 to 83 days (mean duration: 66.56 ± 10.65 days). As part of the aerobic conditioning component of the neurorehabilitation protocol, a subset of Treated-cohort participants (*n* = 6/7; one baseline session was excluded due to an incomplete recording) additionally underwent cardiopulmonary exercise testing (CPET) using a breath-by-breath metabolic analysis system (COSMED K5, COSMED Srl, Rome, Italy) on a cycle ergometer, performed once at the start and once at the end of the aerobic training period. Peak oxygen uptake (VO_2_peak), peak workload (Watt), and metabolic equivalents (METS) were extracted from each session as objective, physiology-based indices of functional exercise capacity. Because CPET equipment availability was limited, the CPET assessment window did not always span the same dates as the blood-draw interval for a given patient; nonetheless, all CPET measurements represent genuine pre- and post-intervention assessments bracketing the training period. Biochemical Analysis and Quantifications: Fasting peripheral venous blood samples were harvested from each subject at two distinct time points: at baseline prior to group assignment (Pre, baseline ‘b’) and immediately following the conclusion of the assigned study period (Post, after ‘a’). Samples were collected via standard venipuncture into vacuum tubes containing clot activators, allowed to fully clot at room temperature for 30 min, and centrifuged at 3000× *g* for 15 min at 4 °C to isolate the serum fraction. The resulting serum was stored in sterile aliquots at −80 °C until final analysis. Quantitative measurement of serum BDNF and SEPP1 concentrations was performed via enzyme-linked immunosorbent assay (ELISA) using high-sensitivity commercial kits (Human SEPP1/Selenoprotein P ELISA Kit, Catalog No. ABIN6962517, antibodies-online GmbH, Aachen, Germany; Human BDNF ELISA Kit, Boster Biological Technology, Pleasanton, CA, USA; Catalog No. EK0307) according to the manufacturer’s instructions. Absolute biomarker shifts (Δ, change) were calculated for each participant using the following standard formula:

$$\Delta \;=\;Post\text{-}intervention\;concentration\left(a\right)-Pre\text{-}intervention\;concentration(b)$$

Statistical Framework: Given the exploratory pilot design and small sample size (N = 16) of this clinical cohort, nonparametric statistical methods were systematically used to avoid reliance on normality assumptions. Inter-group differences in biomarker deviations (ΔBDNF and ΔSEPP1) were analyzed using the two-tailed Mann–Whitney U test. Bivariate correlation dynamics mapping the relationship between individual ΔBDNF and ΔSEPP1 shifts were analyzed using Spearman’s rank correlation coefficient (ρ). All statistical calculations were performed using R (version 4.3.1, R Foundation for Statistical Computing, Vienna, Austria), with significance set at α = 0.05.

## 3. Results

### 3.1. Baseline Patient Characteristics and Demographic Homogeneity

The demographic and baseline clinical metrics demonstrated balanced distributions between the two arms, confirming appropriate cohort homogeneity at intake. The Control cohort (*n* = 9) presented with a mean age of 66.56 ± 11.41 years (comprising 4 males and 5 females), with an etiology of 77.8% ischemic stroke and 22.2% TIA. The Treated cohort (*n* = 7) displayed a mean age of 59.29 ± 6.97 years (comprising 4 males and 3 females), with an etiology of 85.7% ischemic stroke and 14.3% TIA. The primary active comorbidities across both cohorts included chronic hypertension, carotid artery stenosis, type 2 diabetes mellitus, hypercholesterolemia, and stable cardiopathy. The raw clinical and biochemical profiles for all 16 enrolled subjects are detailed in Table 1.

### 3.2. Divergent Serum Biomarker Tracking Kinetics

**Figure 1:**
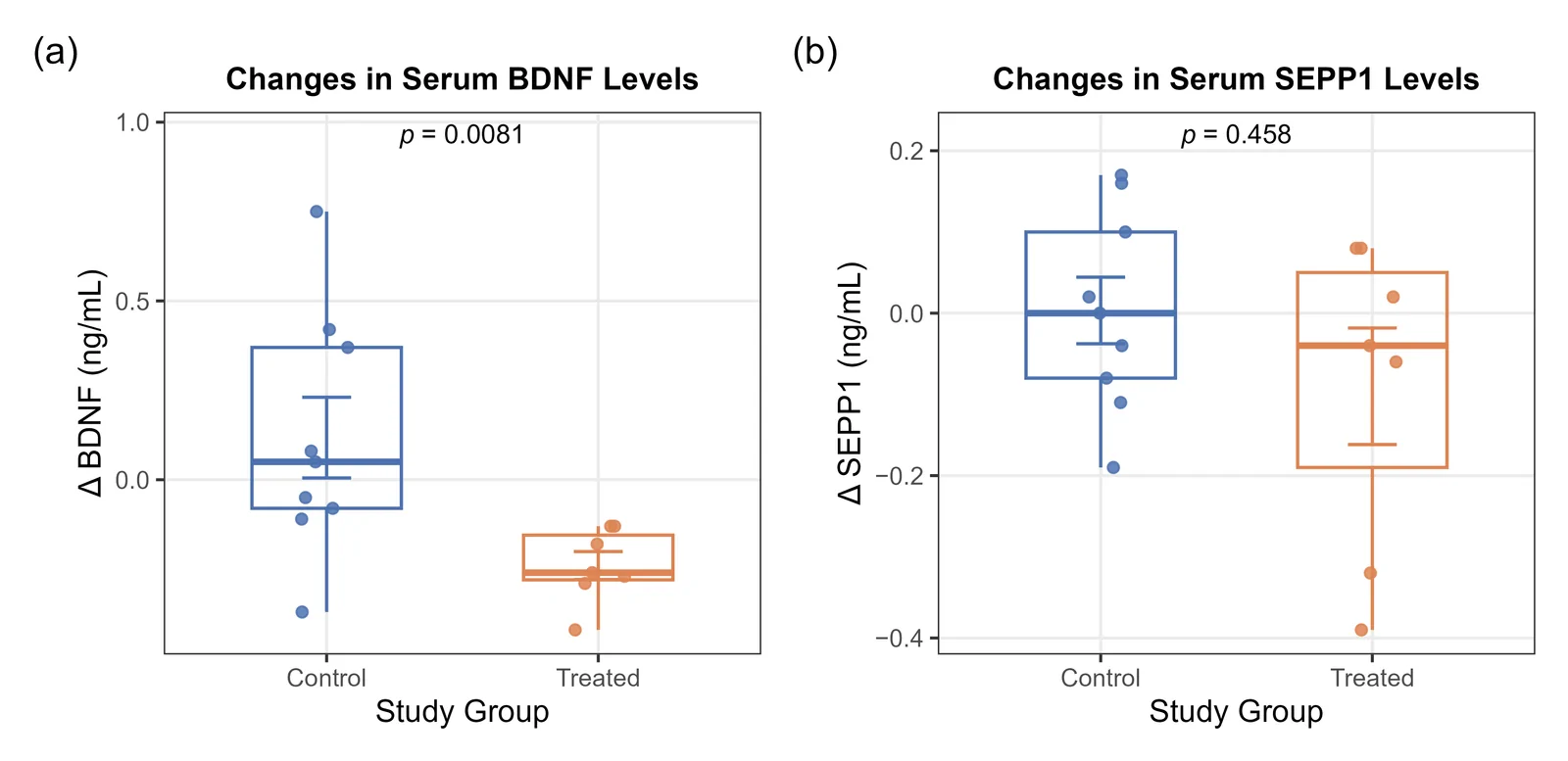
Box-and-whisker plots depicting the absolute changes (Δ) in circulating biomarkers from baseline to post-intervention across the Control and Treated cohorts. (**a**) Significant divergence is observed in serum BDNF levels, with the Treated group showing a uniform decline (Mann–Whitney U test, *p* = 0.0081). (**b**) Changes in serum SEPP1 levels show a descriptive negative trend in the Treated group, though not statistically significant (*p* = 0.458). Individual data points are jittered to display the distribution, and error bars represent the standard error.

Analysis via the Mann–Whitney U test identified a highly robust, statistically significant difference in serum BDNF modifications (ΔBDNF) when comparing the Control and Treated groups (U = 57.0, *p* = 0.0081). The Control cohort displayed variable, non-synchronized neurotrophin changes, presenting a slight descriptive increase or stabilization (mean ΔBDNF: +0.118 ± 0.339 ng/mL). In contrast, the Treated cohort demonstrated a consistent down-regulation of circulating BDNF across all individuals following the multi-modal rehabilitation protocol (mean ΔBDNF: −0.240 ± 0.103 ng/mL) (Figure 1a).

When evaluating serum SEPP1 modifications (ΔSEPP1), no statistically significant difference was observed between the two study arms (U = 39.0, *p* = 0.458). From a descriptive perspective, the Treated cohort exhibited a minor negative trend (mean ΔSEPP1: −0.090 ± 0.190 ng/mL) relative to the stable values observed in the Control group (mean ΔSEPP1: +0.003 ± 0.123 ng/mL) (Figure 1b). Group-level biomarker variations are summarized in Table 2.

### 3.3. Inter-Biomarker Correlation Mapping

**Figure 2:**
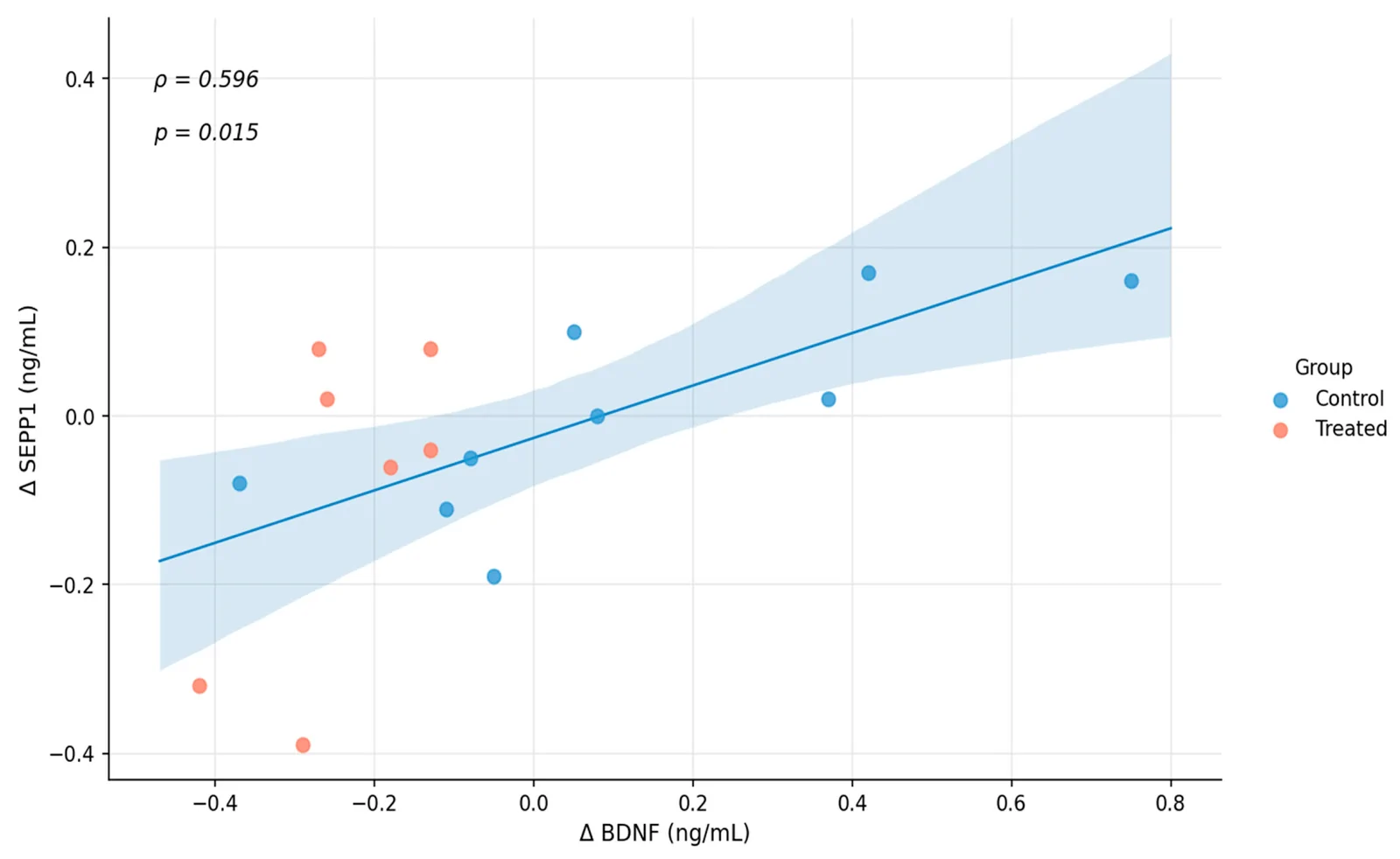
Spearman’s rank correlation analysis illustrating the synchronous biochemical relationship between absolute changes in BDNF (ΔBDNF) and SEPP1 (ΔSEPP1) across the total post-stroke cohort. The strong positive correlation (ρ = 0.596, *p* = 0.015) indicates an integrated parallel dynamic between neurotrophic utilization and antioxidant transport networks during the subacute recovery phase. The shaded area represents the 95% confidence interval of the regression line.

Spearman’s rank correlation testing revealed a significant, strong positive bivariate correlation between individual ΔBDNF and ΔSEPP1 trajectories across the entire post-stroke study cohort (ρ = 0.596, *p* = 0.015). As illustrated in Figure 2, this statistical association indicates a synchronized metabolic response: patients who minimized their peripheral neurotrophin decline or showed minor increases also maintained more stable or elevated SEPP1 concentrations.

As a robustness check, this correlation was also examined separately within each cohort to confirm that the pooled association was not merely an artifact of between-group separation. The correlation remained strong and statistically significant within the Control group alone (ρ = 0.817, *p* = 0.007, *n* = 9) and was directionally consistent, though not statistically significant, within the Treated group alone (ρ = 0.536, *p* = 0.215, *n* = 7), likely reflecting the smaller sample size and narrower range of ΔBDNF values in this cohort. The persistence of a strong within-group correlation in the Control cohort supports the interpretation that the ΔBDNF–ΔSEPP1 association reflects a genuine individual-level physiological relationship rather than a statistical artifact of pooling two distinct groups.

### 3.4. Exploratory Correlation with Functional Outcomes (Post Hoc Analysis)

To further contextualize the biochemical findings, a post hoc exploratory analysis was conducted using cardiopulmonary exercise testing (CPET) data available for a subset of the Treated cohort (*n* = 6/7). Changes in peak oxygen uptake (ΔVO_2_peak) and metabolic equivalents (ΔMETS) between the start and end of the aerobic training period were compared with individual ΔBDNF values using Spearman’s rank correlation. A moderate, negative-trending association was observed between ΔBDNF and ΔVO_2_peak (ρ = −0.543, *p* = 0.266) and between ΔBDNF and ΔMETS (ρ = −0.638, *p* = 0.173), suggesting that participants with a larger decline in peripheral BDNF tended to show greater improvements in aerobic functional capacity. Neither association reached statistical significance, which is consistent with the small available sample size (*n* = 6) for this secondary analysis. These preliminary observations are reported as hypothesis-generating and should be interpreted with appropriate caution; they nonetheless provide a first indication that the observed BDNF dynamics may be physiologically coupled with objective measures of functional improvement, warranting confirmation in larger, adequately powered cohorts.

## 4. Discussion

The principal finding of this pilot study is the significant reduction in baseline serum BDNF levels observed in subacute stroke patients following an intensive, structured neurorehabilitation protocol (*p* = 0.0081). While much of the existing sports-medicine literature focuses on immediate, short-term spikes in circulating BDNF following an isolated session of high-intensity interval training (HIT) due to platelet degranulation [7,8], our data captured a distinct, long-term homeostatic mechanism. This sustained systemic reduction contrasts with the pattern typically reported in moderate-intensity subacute-phase exercise studies (discussed below) and may be partially consistent with the “central sink” hypothesis of peripheral neurotrophin dynamics.

This directional discrepancy merits explicit consideration. Most subacute-phase exercise trials that reported BDNF increases employed moderate-intensity, continuous aerobic protocols; for example, an 8-week program of moderate-to-high-intensity treadmill walking increased serum BDNF by 18% in subacute stroke survivors relative to usual care [11]. The present Treated cohort, by contrast, underwent a long-interval HIT protocol performed at 85–95% of peak-oxygen-uptake-associated heart rate—a substantially higher training intensity. High-intensity exercise is well documented to elevate circulating stress hormones (cortisol/corticosterone), which can blunt or reverse the exercise-induced rise in BDNF typically seen with moderate-intensity training. In a rodent stroke model, high-intensity training produced significantly higher corticosterone levels and no hippocampal BDNF benefit compared with gradually increased or low-intensity training regimens [12]. Consistent with this, intense interval training in young adults has been shown to elevate cortisol and the cortisol-to-BDNF ratio without a measurable increase in circulating BDNF [7]. We therefore consider stress/cortisol-mediated suppression of BDNF release—rather than the central-sink hypothesis alone—a parsimonious and literature-consistent explanation for the decline observed in this high-intensity-trained, subacute cohort. Because this study did not directly measure cortisol or other stress biomarkers, this interpretation remains hypothesis-generating; direct co-measurement of stress hormones alongside BDNF in future, adequately powered trials will be required to test it rigorously.

As a complementary, though less parsimonious, hypothesis, Williams et al. [1] explored these compartments in a similarly sized clinical cohort (N = 7), demonstrating divergent trajectories between serum and plasma BDNF fractions and concluding that serum and plasma BDNF fractions likely reflect different aspects of BDNF regulation. In our Treated cohort, the consistent decline in serum BDNF levels following a multi-week, activity-dependent training program likely reflects an accelerated trans-capillary extraction of the neurotrophin from systemic circulation into the central nervous system. Intensive gait and balance training, ballistic strength exercises, and high-intensity aerobic exercise stimulate prominent cortical reorganization, axonal sprouting, and synaptogenesis within the peri-infarct region [2,6]. This active neuroplastic state creates a massive central metabolic demand for trophic factors, causing cerebrovascular endothelial networks to actively transport circulating BDNF across the blood–brain barrier [13] to sustain central tissue remodeling. It should be noted that the relationship between circulating and central BDNF pools remains incompletely resolved, as experimental stroke models have shown that peripheral and brain BDNF levels do not always correlate directly [14]. This hypothesis is supported by functional in vitro cell models, which demonstrate that serum harvested from stroke patients undergoing aerobic exercise acts as a potent biological driver of neurite outgrowth, dendritic branching, and mitochondrial rearrangement in neuronal lines [8].

Additionally, to our knowledge, this study is among the first to provide clinical evidence of a significant positive correlation between individual changes in BDNF and SEPP1 levels (ρ = 0.596, *p* = 0.015). Selenoprotein P (SEPP1) represents the primary vehicle delivering selenium to the brain and is vital for maintaining cellular redox homeostasis and protecting cerebrovascular endothelial cells from oxidative stress. The synchronized coupling of ΔBDNF and ΔSEPP1 indicates that neuroplastic modifications do not occur in isolation from the local microenvironment. Instead, the central demand for neurotrophic support (BDNF) is functionally linked with antioxidant transport networks (SEPP1) to shield newly forming synaptic configurations from local oxidative damage.

### Limitations

The primary limitation of this study is its small sample size (N = 16), which limits broad generalizability. However, the high statistical significance achieved for BDNF modulation supports its presentation as a Short Communication. This format provides an effective platform to rapidly share clear preliminary trends with the neurorehabilitation community, establishing a foundation for larger confirmatory clinical trials. Additionally, group allocation was not randomized, the follow-up duration differed between cohorts, the subacute study population may have been undergoing substantial spontaneous neurological recovery independent of the intervention (which could itself influence circulating BDNF trajectories), and the exploratory correlation between ΔBDNF and functional exercise capacity (Section 3.4) was based on CPET data available for only a subset of the Treated cohort (*n* = 6); consequently, this association did not reach statistical significance and should be interpreted as hypothesis-generating rather than confirmatory. This caution is reinforced by prior evidence that serum BDNF offers only limited predictive value for post-stroke motor recovery [15]. Additionally, this study did not measure cortisol or other stress biomarkers, precluding direct confirmation of the stress-mediated suppression mechanism proposed above as a candidate explanation for the observed BDNF decline; this represents a specific priority for future studies of high-intensity post-stroke exercise protocols. Larger, randomized studies incorporating standardized clinical and functional outcome measures across both cohorts are needed to establish a definitive link between peripheral biomarker dynamics and rehabilitation efficacy.

## 5. Conclusions

In summary, a structured neurorehabilitation program for subacute stroke patients was associated with a significant decline in serum BDNF concentrations, which may reflect stress/cortisol-mediated suppression of BDNF release associated with the high-intensity training stimulus, although this mechanistic interpretation remains hypothetical and requires direct confirmation. The strong positive correlation with SEPP1 variations suggests a link between neurotrophic signaling and antioxidant pathways, although this correlational finding does not by itself establish a single, coordinated biochemical mechanism. These findings warrant validation in larger clinical trials to correlate biomarker dynamics directly with long-term motor outcomes.

## Figures and Tables

**Table 1:** Comprehensive baseline demographics, comorbidities, and serum biomarker tracking profiles across the study population (N = 16).

Pt ID	Sex	Age (yrs)	Group	Primary Diagnosis	Documented Comorbidities	BDNF b (ng/mL)	BDNF a (ng/mL)	ΔBDNF	SEPP1 b (ng/mL)	SEPP1 a (ng/mL)	ΔSEPP1	Interval (Days)
1	1	60	Control	Ischemic Stroke	Hypertension, Carotid Stenosis	2.39	2.34	−0.05	0.77	0.58	−0.19	83
2	1	73	Control	TIA	Hypertension, Hyperglycemia	0.60	1.02	0.42	0.73	0.90	0.17	62
3	0	86	Control	Ischemic Stroke	Hypertension, Carotid Stenosis	0.46	1.21	0.75	0.61	0.77	0.16	63
4	0	70	Control	Ischemic Stroke	Hypertension	0.89	0.81	−0.08	0.53	0.48	−0.04	63
5	0	65	Control	Ischemic Stroke	Hypertension, Atherosclerosis	1.01	0.90	−0.11	0.65	0.54	−0.11	49
6	1	68	Control	Ischemic Stroke	Hypertension, Carotid Stenosis	0.64	0.72	0.08	0.32	0.32	0.00	81
7	1	43	Control	Ischemic Stroke	None	0.34	0.71	0.37	0.80	0.82	0.02	70
8	0	69	Control	Ischemic Stroke	Hypertension	1.25	1.30	0.05	0.82	0.92	0.10	59
9	0	65	Control	TIA	Hypertension, Cardiopathy	0.87	0.50	−0.37	0.69	0.61	−0.08	69
10	1	57	Treated	Ischemic Stroke	Hypercholesterolemia, Hyperglycemia	1.17	1.04	−0.13	0.77	0.85	0.08	76
11	1	53	Treated	Ischemic Stroke	Type 2 Diabetes, Hypertension	1.31	0.89	−0.42	1.04	0.72	−0.32	76
12	1	69	Treated	Ischemic Stroke	Hypertension, Cardiopathy	1.21	0.95	−0.26	0.85	0.87	0.02	75
13	0	55	Treated	Ischemic Stroke	Bradycardia, Hypertension	1.43	1.30	−0.13	0.90	0.86	−0.04	80
14	1	59	Treated	Ischemic Stroke	Hypertension	0.61	0.43	−0.18	0.84	0.78	−0.06	102
15	0	53	Treated	TIA	Hypertension	1.42	1.15	−0.27	0.45	0.53	0.08	93
16	0	69	Treated	Ischemic Stroke	Hypertension	1.19	0.90	−0.29	1.13	0.74	−0.39	74

Note: In biological sex mapping, ‘0’ represents female and ‘1’ represents male subjects. The terms ‘b’ and ‘a’ denote concentrations captured at baseline and post-interval sessions, respectively.

**Table 2:** Group-level alterations in serum BDNF and SEPP1 concentrations.

Biomarker Parameter	Control Cohort (*n* = 9)	Treated Cohort (*n* = 7)	Statistical Significance (*p*-Value)
Baseline BDNF (ng/mL)	0.939 ± 0.613	1.191 ± 0.278	*p* > 0.05
Post-Interval BDNF (ng/mL)	1.057 ± 0.543	0.951 ± 0.273	-
Mean ΔBDNF (ng/mL)	+0.118 ± 0.339	−0.240 ± 0.103	*p* = 0.0081
Baseline SEPP1 (ng/mL)	0.658 ± 0.157	0.854 ± 0.217	*p* > 0.05
Post-Interval SEPP1 (ng/mL)	0.660 ± 0.204	0.764 ± 0.119	-
Mean ΔSEPP1 (ng/mL)	+0.003 ± 0.123	−0.090 ± 0.190	*p* = 0.458

## Data Availability

The clinical and biochemical source data supporting the findings of this study are available from the corresponding author upon reasonable request, subject to patient privacy and institutional ethical restrictions. The R syntax and analytical data arrays utilized for non-parametric statistical testing are available upon request from the corresponding author.
